# Diel oscillations in cell wall components and soluble sugars as a response to short-day in sugarcane (*Saccharum* sp.)

**DOI:** 10.1186/s12870-019-1837-4

**Published:** 2019-05-23

**Authors:** Leonardo Cardoso Alves, Juan Pablo Portilla Llerena, Paulo Mazzafera, Renato Vicentini

**Affiliations:** 1 Bioinformatics and Systems Biology Laboratory, Department of Genetics and Evolution and Bioagents, University of Campinas, Campinas, SP Brazil; 2 Department of Plant Biology, University of Campinas, Campinas, SP Brazil; 3 Crop Science Department, College of Agriculture Luiz de Queiroz, University of São Paulo, Piracicaba, Brazil

**Keywords:** Sugarcane, Diel, Soluble sugars, Cell wall, Photoperiod, Short-day

## Abstract

**Background:**

Sugarcane is a tropical crop that can accumulate high concentration of sucrose in the stem as a storage carbohydrate. For that reason, sugarcane accounts for approximately 75% of all the sugar produced in the world and has become the main sugar source to produce first-generation bioethanol in Brazil. Daily rhythms cause plants to adapt and coordinate their metabolism to achieve maximum photosynthesis and carbohydrate production throughout the day. Circadian rhythms arise from the interaction of an internal oscillator and external stimuli, whereas diel rhythms occur in response to a light-dark cycle. Diel signalling contributes to synchronizing circadian rhythms to photoperiods, and levels of carbohydrates oscillate in a diel fashion. Under regular photoperiods, they are synthesized during the daytime and consumed throughout the night as an energy reserve. However, short days can induce higher rates of synthesis during daytime and lower rates of consumption in the dark. Cell wall carbohydrates are also diurnally regulated, and it has been shown that celluloses, hemicelluloses and pectin are deposited/degraded at different times of the day. To assess the diel carbohydrate profile in young sugarcane plants, we measured soluble sugars and cell wall components along a time course in plants subjected either to a regular day or short day.

**Results:**

Short-day influenced sucrose synthesis and cell wall components. In short-day a 44% increase in sucrose concentration was detected in the dark, but was stable during the day. Cellulose, hemicellulose and pectin also fluctuate within a 24 h interval when subjected to a short day. A 38% increase in leaf sheath cellulose was observed from the middle of the day to the first hour of the night. Leaf sheath pectin and hemicellulose also increased from the day to the night, while it decreased in leaves.

**Conclusions:**

The presented data show diurnal patterns of soluble sugar metabolism together with temporal regulation of cell wall metabolism for a short day, suggesting that diel signalling has a role in how sugarcane manages sugar accumulation and partitioning. Understanding cell wall synthesis/degradation dynamics may help to improve the yield of sugarcane.

**Electronic supplementary material:**

The online version of this article (10.1186/s12870-019-1837-4) contains supplementary material, which is available to authorized users.

## Background

Sugarcane is a C4 tropical crop that can accumulate concentrations of sucrose as high as 540 mg/g in mature stalks [1]. This characteristic has led sugarcane to be the basis for approximately 75% of the total sugar produced in the world [2], and Brazil harvests approximately 38% of the world’s sugarcane [3]. Additionally, sugarcane is the raw material for biofuel production in Brazil [4]. In 2017, the estimated production of sugarcane was ~ 646 million tons, corresponding to the production of ~ 40 million/tons of sugar and ~ 26 billion/litres of ethanol [5]. Apart from that, sugarcane has attracted interest for the second generation (2G) ethanol industry because of the high rates of biomass production and potential to transform the bagasse into bioenergy [4].

Sucrose, glucose and fructose are the main soluble carbohydrates found in sugarcane leaves and stalks [6, 7]. Two key reactions control sucrose synthesis. First, sucrose-phosphate-synthase condenses UDP-glucose and fructose into sucrose-6-phosphate (S6P), and then, sucrose-phosphate-phosphatase dephosphorylates S6P, resulting in sucrose [8]. In sugarcane, sucrose is rapidly transported from the leaves to the culms for storage through a source-sink mechanism [8–10].

The rotation of the Earth is a 24 h cycle that forces plants to coordinate their metabolism to perform photosynthesis so that carbohydrate metabolism is at maximum power during the light period [11]. Circadian rhythms arise from the control of an oscillator that can run even in the absence of external stimuli, such as light and temperature [11]. However, circadian data are measured by continuous conditions that do not reflect those real conditions plants face [12]. On the other hand, diel rhythms occur in response to a light-dark, or diurnal cycle. Diel rhythms are also major players controlling the response to environmental stimuli, synchronizing endogenous circadian rhythms to external stimuli, such as photoperiods [12].

Sucrose and reducing sugars are diel regulated and differ significantly during the day and night in different light conditions [13]. These carbohydrates are synthesized during the day as photosynthesis products and respired during the night as an energy source. In Sorghum (*Sorghum bicolor*), the starch synthesis pathway is diel controlled [14], whereas Arabidopsis coordinates the amount of starch to be synthesized/consumed according to the length of the day. The shorter the day, the faster starch synthesis is, and the breakdown in the night is slower, so that a minimum level of this carbohydrate is maintained at the end of each night [15, 16].

Recently, a diel profile of sugarcane soluble sugars under regular light conditions was reported, with sucrose being accumulated during the day in leaves and culms and consumed in the night [17]. In sugarcane concentrations of sucrose and reducing sugars have been reported by a broad range of studies, and concentrations of these carbohydrates can largely vary depending on the cultivar, growth conditions and/or stages and tissues [1, 6, 18–27]. However, still there are no reports on how soluble carbohydrates fluctuate in sugarcane cultivars in response to different photoperiods, such as a short-day.

Despite its complexity, the plant cell wall is a dynamic structure composed mainly of cellulose, hemicellulose, pectin, lignin and proteins, and it comprises the major terrestrial carbon reservoir [28, 29]. The wall protects plant cells against pathogens and allows cell expansion by relaxing and shrinking [30]. Sugarcane cell walls from leaves and culms are, like other grasses composed of complex hemicellulose, pectin and pectic arabinogalactans bound to cellulose [31]. Cellulose, a crystalline, inelastic and mechanically resistant material formed by chains of β-1,4 glucose [28, 30], is the main cell wall component. It is embedded in hemicellulose and pectin. Hemicellulose is mostly formed by xyloglucan and arabinoxylan in sugarcane leaves and culms [31] as expected for grasses [28]. Pectin is a matrix made of a glucuronic acid-rich fraction involving hemicelluloses to form a flexible layer over cellulose [28, 30]. The sugarcane pectin fraction comprises approximately 10% of the cell wall [31].

The cell wall changes its composition during plant cell growth, becoming more complex. In different situations, such as fruit softening [32–34], flower development and pollen formation [29, 35], the composition of the cell wall changes dynamically. However, little is known about wall component fluctuation in a smaller time window, such as within one day [36–39], and in response to various environmental stimuli.

Here, we show the first diel dynamic study from soluble sugars and cell wall components under a regular day (RD, 12 h/12 h light/dark) in comparison to a short day (SD, 08 h/16 h light/dark) in sugarcane. Under RD, sucrose was synthesised at a linear rate during the daytime and broken down during the night. However, in SD, a much lower rate of synthesis was measured during the daytime, and surprisingly, a higher rate of degradation was detected during the night. We also demonstrated that cellulose, hemicellulose and pectin are relatively stable during the diel cycle in RD plants. Nevertheless, we showed that a short-day induced significant fluctuations in these cell wall components within one day in sugarcane.

## Results

### Morphological development of the young plants was not affected by short-day

We measured plant height and the length and width of the + 1 leaf at days 0 and 30 from sets of 27 plants under each diel regimen. In addition, dry masses from roots, leaves and leaf sheaths were also measured at those days. At day 0 no significant differences (*p*-value < 0.05) among all the comparisons between RD and SD were detected, suggesting the groups of plants were similar at the beginning of the diel period. Surprisingly, on day 30, again no significant differences (p-value < 0.05) were observed comparing data from RD and SD plants (Table 1 and Additional file 2: Tables S1-S4). This suggests that the different light regimes had no influence on the morphology measured of young sugarcane plants.

**Table 1:** Morphological measurements from plants under each diel regime on days 0 and 30

	+ 1 leaf		Plant height (cm)	Dry mass			
	Length (cm)	Width (cm)		Leaves (g)	Leaf sheath (g)	Roots (g)	Total (g)
12 h/12 h light/dark							
Day 0	37.82 ± 12.4	1.09 ± 0.2	53.44 ± 14.8	0.53 ± 0.3	0.72 ± 0.4	0.6 ± 0.4	1.85 ± 1.0
Day 30	38.17 ± 12.3	1.1 ± 0.2	85.03 ± 25.3	1.01 ± 0.5	0.94 ± 0.5	0.57 ± 0.4	2.52 ± 1.3
08 h/16 h light/dark							
Day 0	37.49 ± 12.3	1.18 ± 0.2	56.21 ± 16.1	0.43 ± 0.3	0.58 ± 0.3	0.39 ± 0.2	1.4 ± 0.8
Day 30	38.79 ± 11.6	1.2 ± 0.2	91.68 ± 25.3	0.9 ± 0.4	0.85 ± 0.2	0.46 ± 0.2	2.21 ± 0.8

The dry masses from leaves, leaf sheaths and roots from plants under each diel were measured at day 0 and from other set of plants at day 30. Values are mean ± SD where *n* = 27 for + 1 leaf length and width and plant height; and n = 3 for dry mass at day 0 and 30

### Concentration of cell wall components fluctuate when subjected to a short-day regime

**Fig. 1:**
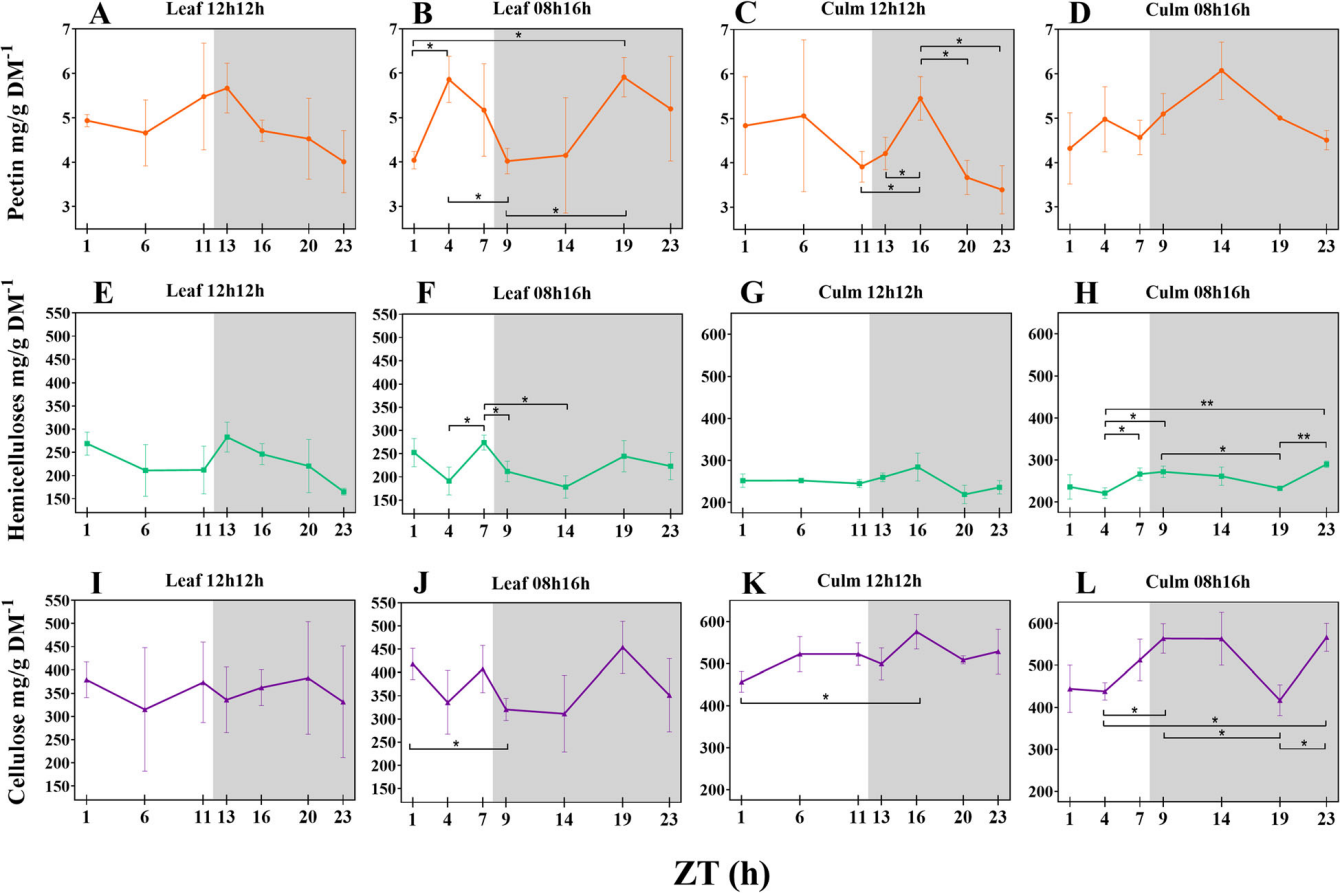
Diel fluctuation of cell wall components in response to photoperiods (**a-l**). Differences within means from each timepoint under each photoperiod and tissue were accessed by t-test. Bars connect significantly different timepoints. * *P* < 0.05; ** *P* < 0.01. Each point represents the mean ± SD (*n* = 3). Daytime (white) and night (light grey)

We quantified the cell wall fractions in leaves and leaf sheaths from RD and SD plants (Additional file 2: Table S5). Hemicellulose comprised ~ 24% of cell wall dry mass in both tissues and photoperiods, and pectin corresponded to ~ 5%. Cellulose comprised ~ 50% of leaf sheath cell wall dry mass and ~ 37% in the leaf. There was a tendency for variation in concentrations during the day, such that the proportions of each component relative to the dry mass and in response to photoperiod varied (Fig. 1). Most of these variations were observed in SD plants. Nevertheless, leaf sheath pectin and leaf sheath cellulose from RD plants also underwent changes during the 24 h period, mainly in the night. In SD plants, all the components oscillated in leaves, and hemicellulose and cellulose also oscillated in the leaf sheaths. As an illustration, leaf sheath cellulose from SD plants increased from 438.15 mg/g DM^− 1^ in the middle of the day (Zeitgeber time 4 - ZT4) to 563.54 mg/g DM^− 1^ at the beginning of the night (ZT9; *p*-value 0.0194) and to 566.40 mg/g DM^− 1^ at the end of the night (ZT23; *p*-value 0.0151). Leaf hemicelluloses rose from 191.01 mg/g DM^− 1^ at ZT4 to 273.86 mg/g DM^− 1^ at the end of the day (ZT7; *p*-value 0.0404); and from ZT7 to ZT9 it was reduced by 62 mg/g DM^− 1^ (*p*-value 0.0368). Additional file 2: Table S6 shows significant (*p*-value < 0.05) fluctuations of cell wall components during the 24 h period.

In plants subjected to RD the distribution of the components was stable in the daytime. Interestingly, in leaves of SD plants the average pectin concentrations showed an opposite pattern compared to cellulose and hemicelluloses during the daytime, peaking from the middle of the day. In SD plants the pectin rapidly increased by 45% from ZT1 to ZT4 (*p*-value 0.0263), while cellulose declined 23% from ZT1 to ZT9 (*p*-value 0.0331) (Fig. 1b, f, j).

Leaf sheath pectin and cellulose from RD plants peaked at ZT16 (Fig. 1c, k), with a drastic pectin increase from the end of the day to ZT16 (+ 39%, *p*-value 0.0267). Cellulose content increased by 26% from ZT1 to ZT16 (p-value 0.0341) (Fig. 1k). In SD plants, a different distribution of the averages for each leaf sheath component was detected in comparison to RD plants, as was also observed in the leaves. Pectin peaked at the same time period in RD, whereas cellulose peaked at the day-night transition and was the same at ZT14 (Fig. 1l). Except for ZT19, leaf sheath cellulose maintained stable concentrations during the night and showed an inverse pattern of distribution in comparison to leaves in the night (Fig. 1j, l). At ZT19, average pectin and cellulose concentrations in the leaf sheath had fallen, whereas this was the peak time in the leaves. Hemicellulose increased during the day and decreased during the night, as observed in leaves. However, hemicellulose peaked in ZT23 and, like with cellulose, most of the significant differences were between ZT4 and the timepoints in the night (Fig. 1h).

### A short-day stabilizes sucrose synthesis during the day and induces synthesis during the night in sugarcane leaves

**Fig. 2:**
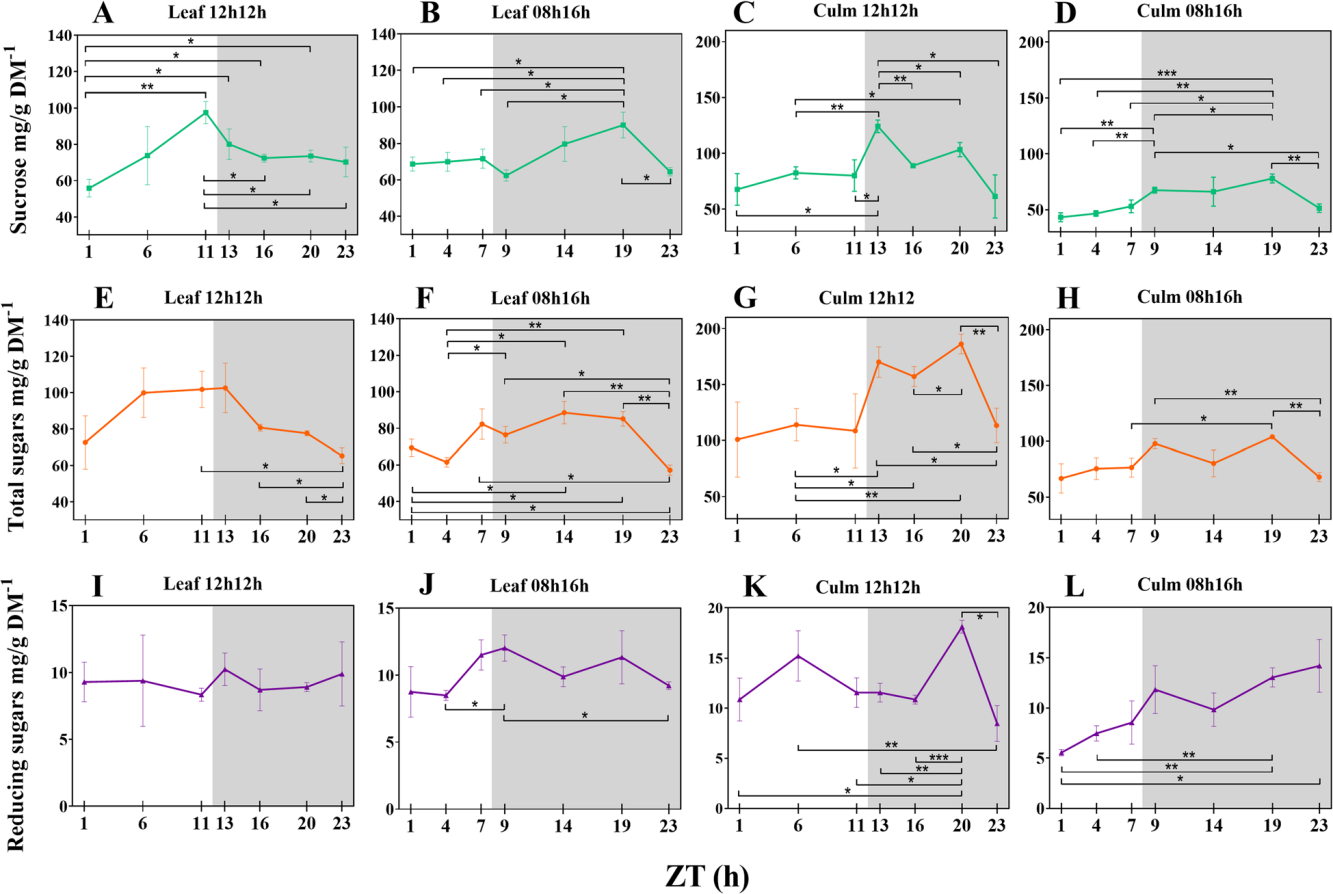
Diel fluctuation of soluble sugars in response to photoperiods (**a-l**). Differences within means from each timepoint under each photoperiod and tissue were accessed by t-test. Bars connect significantly different timepoints. * *P* < 0.05; ** *P* < 0.01; *** *P* < 0.001. Each point represents the mean ± SD (n = 3). Daytime (white) and night (light grey)

We quantified the total soluble sugars, sucrose and reducing sugars composition of leaves and leaf sheaths during the time course (Additional file 2: Table S7). Our data showed that synthesis of sucrose and total sugars in the leaves and that storage of all the fractions were higher in RD plants (Fig. 2). Additional file 2: Table S8 shows significant fluctuations in soluble sugars throughout the 24 h period. Leaf sucrose was linearly accumulated during the day and increased ~ 75% in concentration from the beginning to the end of the day (*p*-value 0.0020; Fig. 2a), whereas in the leaf sheaths, it peaked right in the first hour of the night (ZT13; Fig. 2c). Sucrose is transported from leaves to leaf sheaths and fluctuated from ~ 80 to 124 mg/g DM^− 1^ (55 + %) in the two-hour interval comprising the day-night transition (ZTs 11–13; *p*-value 0.0338). In contrast, leaf sheaths from SD plants did not significantly alter their sucrose content during the same transition (ZTs 7–9; ~ 53 to ~ 67 mg/g DM^− 1^; Fig. 2d). The most drastic differences in leaf sucrose concentrations were detected at ZT1 vs ZT11 (p-value 0.0020) and ZT1 vs ZT13 (p-value 0.0355) under RD. SD plants behaved in a different fashion regarding sucrose concentration dynamics. They accumulated much less sucrose than RD plants during the day (Fig. 2b, d). In the night, sucrose notably accumulated in the leaves until ZT19 and the concentrations in leaves and leaf sheaths were similar (Fig. 2b). Due to these dynamics, a ~ 44% increase in sucrose concentration was detected in the comparison between ZTs 9 and 19 (p-value 0.0186), the largest difference observed in SD plants.

The total sugars fraction also increased during the day and decreased during the night similarly to sucrose in all tissues and photoperiods because sucrose is synthesized at much higher concentrations than the other sugars (Fig. 2e, f, g, h). However, in RD plants leaves, the only significant changes in concentrations of sucrose were detected from ZT11 (Fig. 2a). Additionally, in leaf sheaths, the pattern of distribution of total sugars and sucrose was very similar. Concentrations were quite stable during the day and then increased from the transition between day and night to a peak at the first hour of the night. They then tended to decrease in concentration and reached another peak at the penultimate time point (Fig. 2c, g).

The reducing sugar load in the leaves of SD plants fluctuated. Although they were stable in RD leaves during the period (Fig. 2i), an increase was detected from ZT4 to ZT9 (+ 42%, *p*-value 0.0248) when under 8 h of light (Fig. 2j). During the night, the concentration decreased 23% from ZT9 to ZT23. A different distribution was observed in the leaf sheaths in response to each photoperiod. Under regular illumination, a peak was reached at ZT6 and another at ZT20 (Fig. 2k), the last accounting for double the leaf concentration. SD plants accumulated fewer reducing sugars in the leaf sheath than in the leaf during the day. Nevertheless, these sugars increased from ZT1 to ZT23 (+ 156%, p-value 0.0416) (Fig. 2l), when they were also higher than the leaf content (p-value 0.0312).

## Discussion

We found no morphological differences between sugarcane under RD and SD regimes. This is inconsistent with observations of 31 days-old Arabidopsis growing under 08 h/16 h L/D, whose growth rates were slower than under 12 h/12 h L/D [40]. However, the photoperiod length (in hours) does not necessarily play an exclusive role in growth; the number of days of the photoperiod treatment may also be considered. A 21 days short-day treatment had no effect in leaf area, petiole length or yield, whereas 35 days of treatment resulted in lower rates in strawberry [41]. Additionally, young sugarcane grows slower than other crops until it reaches the stem elongation stage [42]. This suggests the shortened light period had no influence on the dry mass, plant height, and + 1 leaf length and width of young sugarcane.

**Fig. 3:**
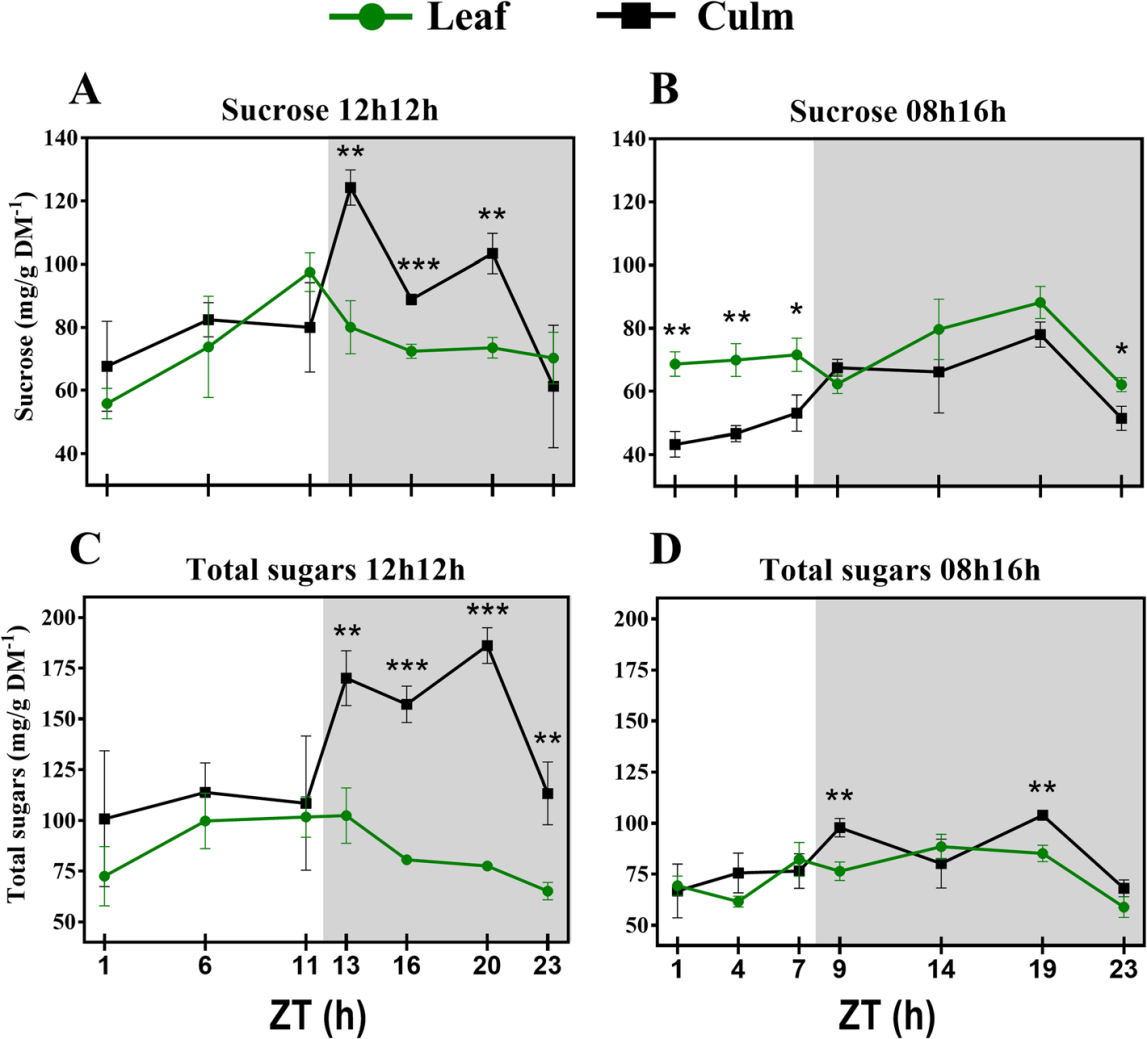
Diel fluctuations of soluble sugars (**a-d**) in leaves and leaf sheaths under each photoperiod. Each point represents the mean ± SD (n = 3). Daytime (white) and night (light grey). Student’s t-test was performed for comparisons of means within each time point, where the significant levels were determined as * *P* < 0.05; ** *P* < 0.01; *** *P* < 0.001

We also measured soluble sugars from sugarcane. The data agree with several other studies that have reported soluble sugars measurements (Additional file 2: Table S9). We reported an adjusted source-sink mechanism in plants under a regular photoperiod. RD plants synthesized sucrose during the day by photosynthesis, and translocated sucrose from leaves to leaf sheaths during the night. During the day, leaves and leaf sheaths had similar amounts of sucrose, whereas in the night, much more sucrose was found to have accumulated in the leaf sheaths (Fig. 3a). These data agree in part with a recent report on diurnal fluctuation of soluble sugars in field-grown sugarcane [17], where sucrose accumulated in leaves of 3-month-old plants from the middle to the end of the day, but no significant differences were shown in the night [17]. In the present study, shortening of the light period to only 8 h resulted in adjustments on this dynamic. Sucrose concentrations in the leaves were very stable through the day, nevertheless still much higher than in the leaf sheaths (Fig. 3b). During the night, the leaves and leaf sheaths accumulated sucrose in a similar pattern as the RD plants. The source-sink mechanism is also supported at a transcriptional level. As previously reported, leaf phase transcript profiles (phase being the time of the day when expression of a given gene reaches a peak) for enzymes associated with sucrose metabolism coincide with these dynamics. Genes related to sucrose synthesis are highly induced in the early morning, whereas genes related to sucrose breakdown are more highly induced during the night [43].

Concentrations of total sugars were higher in leaf sheaths and fell from ZT 13 to 23 in RD in this organ and from ZT11 to 23 in the leaves (Fig. 3c), similar to sucrose at night. During daytime the amounts of total sugars were the same in leaf sheaths and leaves. In SD only ZTs 9 and 19 were significantly different between leaf sheaths and leaves. The pattern from RD plants was consistent with a previous study in rice leaves [44]. Such responses might be triggered by photosynthesis in rice, as expression of genes is mostly phased in the beginning of the day (ZT1) to assure sugar synthesis. In addition, their peaks correlate with the phasing of transcripts for light harvesting complexes reported in sugarcane under a constant light regime [43]. In Arabidopsis, photosynthesis is under circadian control [45], however circadian-regulated genes also show a diel expression pattern [46]. In rice, under natural conditions, a diel influence together with circadian control has also been reported [44]. Authors also reported that the physiological photosynthetic apparatus was also regulated by the diel cycle together with many genes for light harvesting complexes that phase at the first hour of the day or at the first hour and the middle-end of the day.

**Fig. 4:**
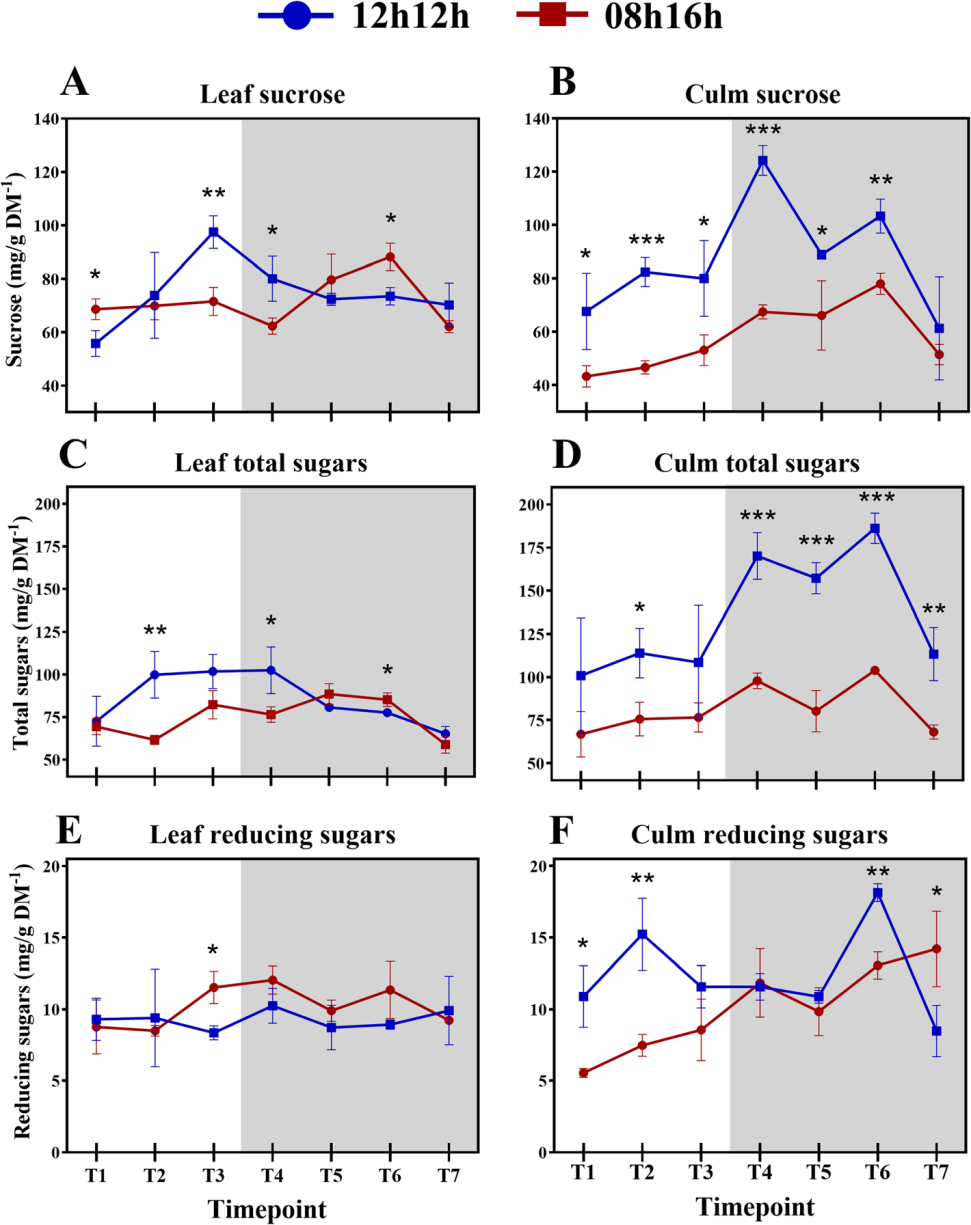
Diel fluctuations of soluble sugars (**a-f**) between plants subjected to a 12 h/12 h and 08 h/16 h light regimes. Each point represents the mean ± SD (n = 3). Daytime (white) and night (light grey). Student’s t-test was performed for comparisons of means within each time point, where the significant levels were determined as * *P* < 0.05; ** *P* < 0.01; *** *P* < 0.001

Sucrose is transported to the sink when at higher concentrations, which means less sucrose in the source could lead to less sucrose being available to export [47]. Indeed, sucrose accumulation in SD plants was significantly lower than in RD plants (Fig. 4a), which may be the reason for the difference between the source and sink. Consistent with this interpretation, in Arabidopsis a 08 h/16 h photoperiod results in a lower amount of total starch accumulation than a 12 h/12 h photoperiod [15, 40]. In Arabidopsis, it has been shown that short days induce faster starch synthesis during the day and subsequently lower consumption during the night [15] in a mechanism that can be mathematically modelled [16] to maintain the necessary amount of this storage carbohydrate. That did not occur in leaves from SD sugarcane plants. In sugarcane, sucrose is the storage carbohydrate, and during the day, sucrose concentration did not oscillate. Surprisingly, during the night, sucrose in the leaves increased from ZT9 to ZT19, even though there was no photosynthesis. From ZT19 to 23, the accumulation fell to a level close to ZT1. Interestingly, the concentration of cell wall components tended to peak at ZT19 in the leaf (Fig. 1b) and notably only under SD where there were significant differences between time points from the day vs night regarding to cell wall components (Additional file 2: Table S6).

Altogether, these data suggest that what normally occurs in sugarcane during the day in a regular photoperiod was shifted to the night to alleviate the lack of light exposure in SD plants. This mechanism, however, was still not sufficient to synthesize and store sucrose and total sugars at the same amounts detected in RD plants because of the shorter day (Fig. 4b, d). A sugar-signalling pathway might play a role in this metabolic reorganization and influence the sugar accumulation capacity. In fact, carbon-responsive genes showed accentuated diurnal changes as well as many circadian-regulated genes in Arabidopsis [46]. In the starchless Arabidopsis mutant *pgm* the low levels of glucose at the end of night induced many glucose responsive genes suggesting sugars play a significant contribution to the diurnal regulation of metabolism [46]. In sugarcane a set of genes associated with sucrose content and related to signal transduction were found to be early sugar-responsive [48]. More recently, signal transduction genes and a SnRK-interacting protein were shown to be enriched in a high-Brix sugarcane cultivar [49], reinforcing this hypothesis.

Generally, leaf sheaths and leaves from SD plants accumulated much less sucrose and total sugars during the 24 h period (Fig. 4a, d). These observations suggest that sucrose breakdown and transport to the leaf sheath was accentuated in SD plants to prioritize sucrose accumulation in that tissue. That makes sense, as the growth pattern of sugarcane is optimized to increase sucrose content [42].

It is also possible that other sugars might have been reallocated to nocturnal sucrose synthesis besides the already available glucose and fructose. Indeed, there were no differences in the concentrations of reducing sugars in RD leaves, in accordance with a recent report for glucose and fructose [17]. On the other hand, SD leaves have accumulated reducing sugars from the middle of the day (ZT4) until the beginning of the night (ZT9) (*p*-value = 0.0242; Fig. 4e, f). In addition, differences in the nocturnal concentrations of total sugars between leaves and leaf sheaths were much smaller in SD than RD plants (Fig. 3c, d), indicating that these sugars could have been allocated to the leaf as substrates for sucrose synthesis, as there was no photosynthesis occurring at the time. Maltose (a reducing sugar), triose phosphate and glyceraldehyde 3-phosphate are products of starch degradation and can be utilized as substrates and regulators for sucrose synthesis in Arabidopsis [50, 51]. On the other hand, it has been shown that the starch concentration is too low, smaller than 1.6 mg/g DM^− 1^, in the sugarcane cultivar reported in the present study (SP83–2847) [52]. Because of the importance of sucrose to sugarcane, the short-day condition influenced the synthesis of sucrose at night until ZT19 to maintain a reasonable storage of this carbohydrate. Sucrose then returned at ZT23 to a concentration similar to ZT1.

Further studies on gene expression could shed light on genes and pathways involved with these responses. It has been shown sugar transporters play role on phloem loading of sucrose [53] and collaborate to control source-sink mechanism [47] in Arabidopsis [54] and sugarcane [49, 53]. The power of combined large-scale transcriptomics, protein measurements and kinetic modelling could collaborate to build more realistic models that addresses perturbations [55] in a diurnal fashion, such as different photoperiods. Little is known on the regulation of sugar transporters during stress because most of the studies are conducted at a physiological levels [47], therefore understanding that mechanism may led to higher yields in response to diverse environmental conditions.

The fluctuations of cell wall carbohydrates also attracted our attention. Because (i) cell wall components contribute to the maintenance of the mechanical properties of the wall and (ii) cell wall depends on carbohydrate biosynthesis, we attempted to measure the concentrations of wall components at each time point to generate knowledge about photoperiod control of wall metabolism during a 24 h period.

Cell wall assembly/disassembly dynamics allow cell to relax and stretch without destroying the wall during development. Polysaccharides are deposited and modified according to the needs of a plant to adapt to the environment [29, 56]. In wheat seeds, genes responsible for synthesis/degradation of cell wall components are expressed at different stages of development, demonstrating these carbohydrates are synthesized constantly [57]. Cell walls from grains, leaf sheaths and leaves of the grass *Brachypodium distachyon* and sugarcane cell suspension cultures contain high amounts of glucosyl hydrolases (GH), oxido-reductases (OR) and invertases (INV) [58–60]**.** GH and ORs (mainly peroxidases) play roles in the maintenance of the polysaccharide networks, allowing the assembly of hemicelluloses and pectin components [59] and changes in hemicelluloses composition through time [56]. INV disassembles the components to ensure the presence of precursors for the synthesis of other cell wall components [58]. In general, the assembly/disassembly of the cell wall seems to have a regular behaviour during development in normal conditions.

**Fig. 5:**
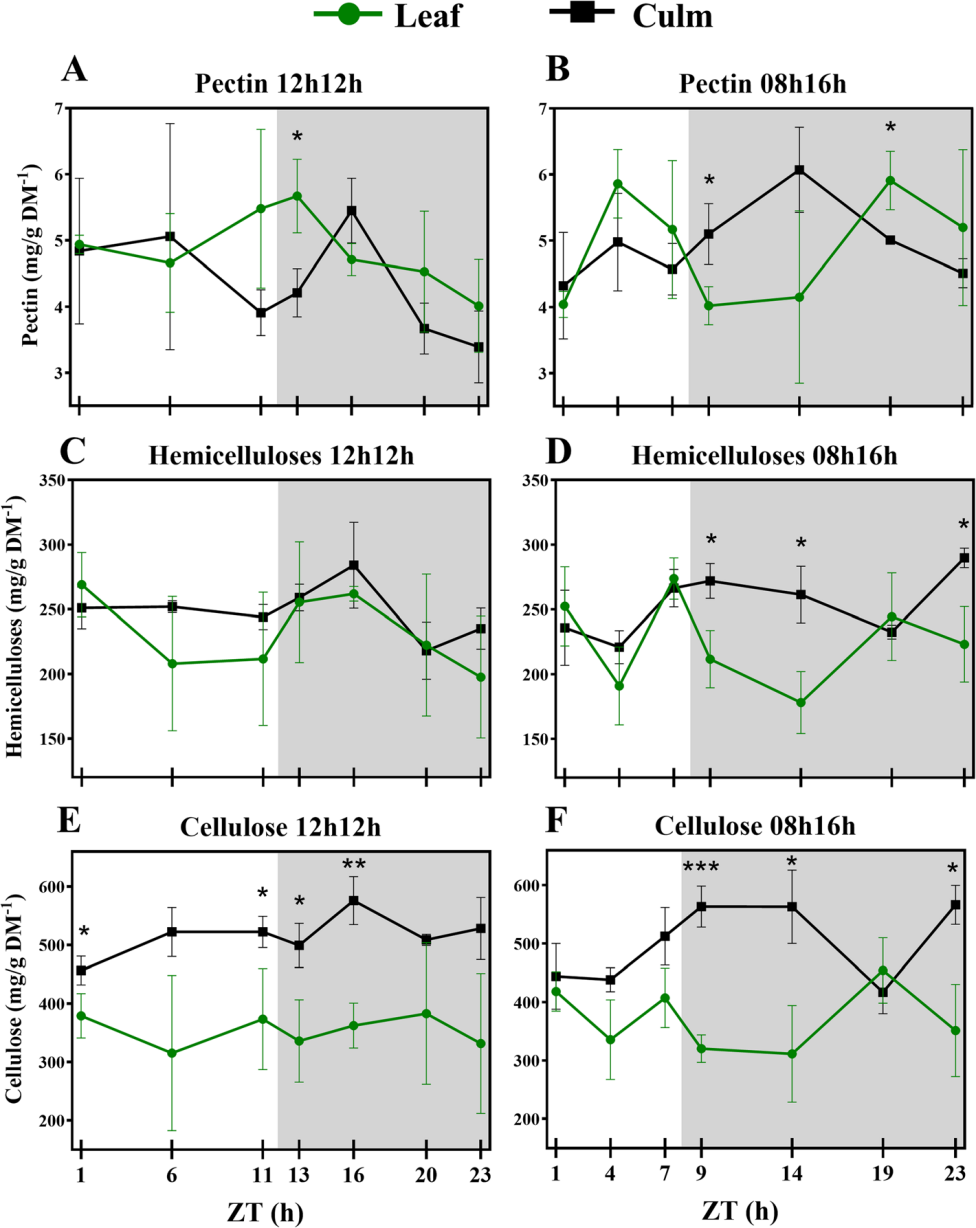
Diel fluctuations of cell wall components (**a-f**) in leaves and leaf sheaths under each photoperiod. Each point represents the mean ± SD (n = 3). Daytime (white) and night (light grey). Student’s t-test was performed for comparisons of means within each time point, where the significant levels were determined as * *P* < 0.05; ** *P* < 0.01; *** *P* < 0.001

A few studies have demonstrated the diel effect on the cell wall metabolism of trees [36–39]. However, neither young grasses nor the photoperiod effect have been the focus of those studies. [36, 37]. A diurnal transcriptome of *Eucalyptus* sp. xylem shed light on wall diel dynamics [39]. Differentially expressed genes phased the most at ZT16, the same ZT where we found the major peaks in RD wall components in the present study (Fig. 5a, c, e). Diel control was reported for some wall formation genes, namely sucrose synthase and UDP-glucuronic acid decarboxylase. Meanwhile, a diurnal pattern of wood formation was reported in *Populus* sp. by tracking radioactive CO_2_ incorporation into all wall components [38]. The authors reported the highest ^13^C incorporation into cell walls happened at night. A negative correlation between hemicellulosic sugars and night has also been reported, suggesting that hemicellulose deposition do not take place fully in the dark.

**Fig. 6:**
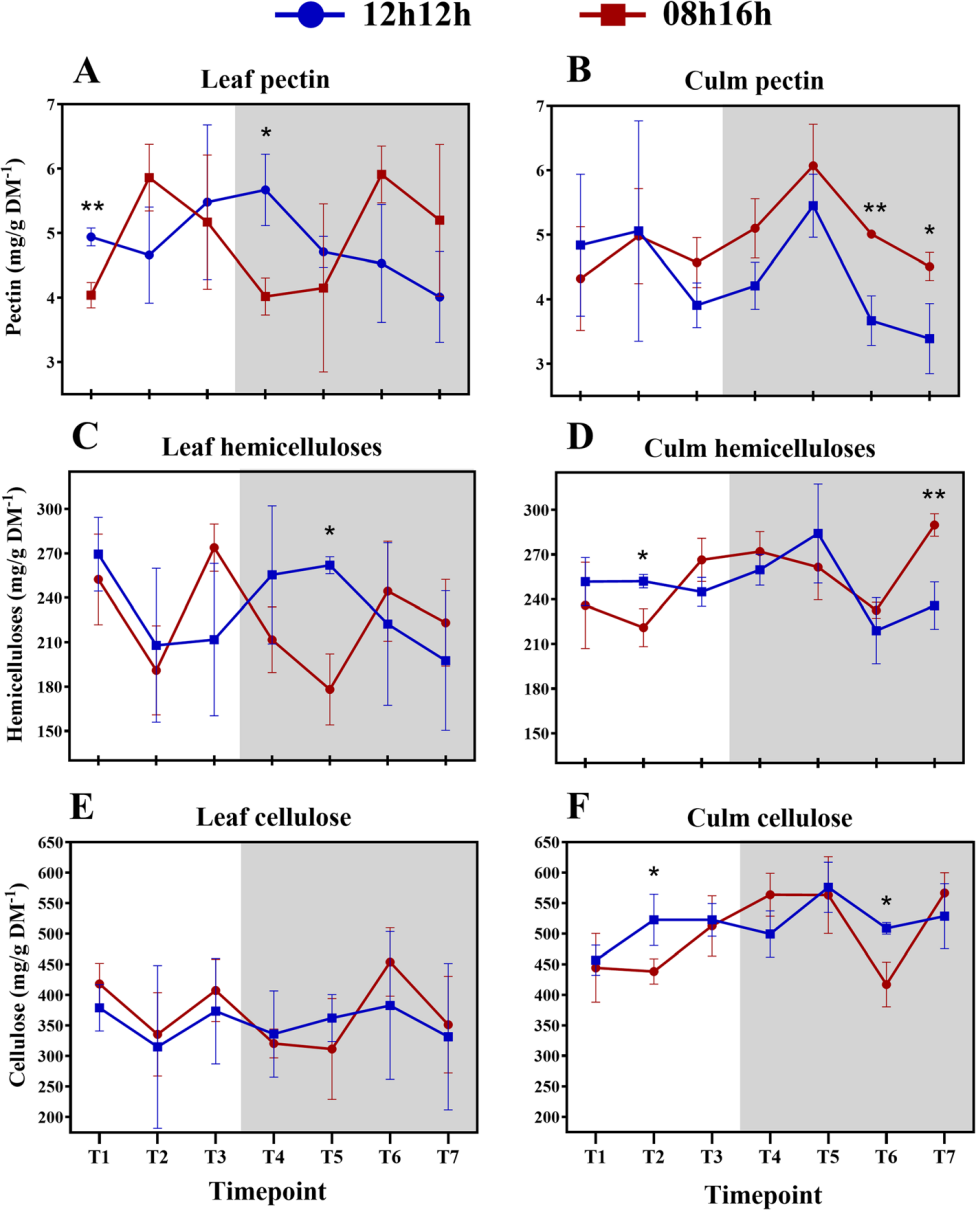
Diel fluctuations of cell wall components (**a-f**) between plants subjected to a 12 h/12 h and 08 h/16 h light regimes. Each point represents the mean ± SD (n = 3). Daytime (white) and night (light grey). Student’s t-test was performed for comparisons of means within each time point, where the significant levels were determined as * *P* < 0.05; ** *P* < 0.01; *** *P* < 0.001

Cellulose, hemicelluloses and pectin compose approximately 95% of young sugarcane cell walls [31, 61], which encouraged us to measure those fractions. After 30 days of being subjected to an SD light regime, the concentrations of cell wall components from the sugarcane cultivar were about the same as RD, indicating no effect from the photoperiod that may have resulted in differential thickness of cell walls during the 24 h period (Fig. 6a-f). Indeed, the rates of each cell wall fraction were similar to previous reports [31, 62], suggesting a level of stability in these concentrations across cultivars. In RD plants, there was no significant fluctuation (*p*-value < 0.05) of cell wall components in the leaf during the period, and only punctual differences were detected in leaf sheath pectin and cellulose (Fig. 1c, k; Additional file 2: Table S6). Additionally, no differences in measured morphological characteristics were observed after the photoperiod treatment, as discussed above. Taken together, these observations suggest that young sugarcane SP83–2847 cultivar is weakly affected in cell wall metabolism by photoperiods when subjected to a regular day. Cell wall metabolism in developing sugarcane tends to behave in a linear fashion during diel cycles under such conditions, except for pectin. Short-day, however, influenced fluctuations on the concentrations of wall components. All but leaf sheath pectin significantly fluctuated (p-value < 0.05) during the 24 h period (Fig. 1b, d, f, h, j, l; Additional file 2: Table S6).

Cell walls from leaves and leaf sheaths are quite similar in composition, but differ in the fine structure of pectin and hemicellulose [31]. The focus of the present study was not to analyse the fine structure of the walls but to investigate fluctuations in the general components, and their proportions matched previous reports [31, 62]. Leaf sheath and leaf components fluctuate similarly during the day in RD plants. Pectin significantly increased and decreased during the night in the leaf sheath and tended to decrease in the leaves as well. The only differences among those tissues were detected in the cellulose fraction. The early sugarcane developmental stages take place before the proper stem elongation stage [63], a leaf sheath structure is already present and it accumulated significantly more cellulose than leaves blades in RD and SD (Fig. 5e, f).

Cell walls are sometimes referred as static structures, even though they are composed of a large amount of sugars which can be a source of energy for growing plants [29]. In addition, plants are able to recycle primary cell wall components [29] during development and during a single day. Like soluble sugars, SD induced fluctuations in cell wall components. The reason that sugarcane assembles/disassembles its cell walls within a day relies on two assumptions: (1) that it can be a source of sucrose synthesis precursors and/or (2) that it may be related to cell and tissue growth.

The second assumption does not seem to be clearly supported by the present data because plant growth was not influenced by the photoperiod. Even so, differential fluctuations were mostly detected in the SD plants (Additional file 2: Table S8). Therefore, fluctuations in pectin and hemicelluloses might play a role as a source of sucrose synthesis precursors. Accordingly, as leaf sucrose increased during the night, pectin and hemicellulose content decreased (Fig. 6a, c). Different trends were seen for pectin in leaf and leaf sheath. In leaves, pectin peaked during the day and night, whereas in leaf sheaths, they tend to accumulate in the middle of the night. The major part of sugarcane pectin is water soluble and not linked to other wall components [31] and so it is easier to reallocate, which could explain the rapid increases/decreases. Several pectin breakdown genes were up-regulated in a high-biomass sugarcane genotype, reinforcing the suggestion that pectin is rapidly degraded for recycling [64].

The patterns of hemicellulose and cellulose distribution were similar in the leaf and leaf sheath fractions under RD and SD (Fig. 1f, g, j, k). Previous transcriptomic data showed that UDP-glucuronic acid decarboxylase, a key enzyme in hemicellulose synthesis, shared a very similar pattern of expression with Sucrose Synthase [39], which is linked to cellulose biosynthesis [64]. Coordination of hemicellulose and cellulose might be advantageous, as xylan functions as cross-linking matrix holding cellulose microfibrils [39].

The cellulosic fractions fluctuated interestingly in the present study. Under RD, a peak in ZT16 was seen in the leaf sheaths; nevertheless, the only significant difference within the diel was between ZTs 1 and 16 (Fig. 5e; Additional file 2: Table S6). On the other hand, another pattern for cellulose deposition was found in SD plants. The cellulose concentration in leaves fell down from ZT1 to ZT9. Meanwhile, cellulose in leaf sheaths increased from the middle of the day to the first hour of the night and then reached a plateau until falling at ZT19 and recovering to the same value as the rest of the night (Fig. 5f). Curiously, at ZT19, leaf and leaf sheath cellulose presented the same concentration. The same was seen for hemicelluloses (Fig. 5d). Most of the literature data for the diel cellulose deposition comes from trees stems [36–38], as discussed above. Our results from SD plants are similar to those previous results: cellulose was deposited mainly from the afternoon to the night. A decrease in cellulose content at ZT19 could mean that sugar turnover took place in the leaf sheaths to contribute to sucrose synthesis in the leaf. Supporting this hypothesis is the fact that most of the differences in cellulose concentration in the leaf sheath during the diel were between a day and night (Additional file 2: Table S6).

## Conclusions

Knowledge of wall dynamics for a given time interval of a day in response to a short-day regime can collaborate to understanding how sugarcane accumulates sucrose and cell walls. In this study, we surveyed the levels of soluble sugars and cell wall components in developing sugarcane under diel control. We concluded that sugarcane plants adapt to short days and metabolize the cell wall in a different fashion while maintaining a reasonable amount of sucrose through nocturnal synthesis.

## Methods

### Plant material and photoperiodic conditions

The present study aimed to report the diel carbohydrate profile in sugarcane cultivars when subjected to regular and short-days; and to evaluate the influence of such regimes in the soluble sugars and cell wall components along a time course in those plants.

One hundred and twenty-four plants from the commercial sugarcane variety SP83–2847 (*Saccharum sp*) were kindly donated by the *Centro de Tecnologia Canavieira* (CTC), Piracicaba, Brazil. Plants were propagated by stem cuttings and grown in small pots (5 cm × 5 cm × 6.5 cm; 160 cm^3^) under greenhouse conditions at CTC. After 60 days, when the plants were ~ 40 cm high, plants were divided into two groups and entrained to different photoperiods for 30 days in a controlled chamber. Light was supplied by light-emitting diodes under a 12 h light (100 μmol m^− 2^ s^− 1^ photon flux density [43])/12 h dark (regular day, RD) or 8 h/16 h light/dark (short day, SD), the temperature was maintained at 27 ± 2 °C and the CO_2_ concentration inside of the chamber was 405 ppm. Light and CO_2_ concentration were measured using an Infra-Red Gas Analyser LCPro+ (ADC Bioscientific). Plants were watered twice a day and their positions were randomly reorganized inside the chamber every week. After the 30 days (90 days-old plants) the leaf sheaths (a structure made of multiple leaves accumulating sucrose) and the middle section of + 1 leaves (the uppermost leaf that has a visible dewlap [63, 65]) with the middle vascular tissue removed were harvested from six (RD) and four (SD) plants at each of the following time points during a period of 24 h (ZT0 means the moment – in hours – after the lights were turned on): ZT1, ZT6, ZT11, ZT13, ZT16, ZT20 and ZT23 for the 12 h/12 h light/dark cycle; and ZT1, ZT4, ZT7, ZT9, ZT14, ZT19 and ZT23 for the 8 h/16 h light/dark cycle. Thus, ZT1, 6 and 11 in 12 h/12 h (RD) and ZT1, 4 and 7 in 8 h/16 h (SD) corresponded to 1 h after starting illumination, the middle, and the last hour of the light period, respectively. The other ZT periods corresponded to the first hour after darkness (ZT13 in RD and ZT9 in SD) and then two points within the dark period and the last hour of the darkness period (Additional file 1: Figure S1). All the material was frozen under liquid nitrogen and stored at − 80 °C until further analysis.

Plant height (root-shoot transition to the tip of youngest leaf (at the top of the plant), + 1 leaf length and width (from the widest part) were measured for 27 plants at 0 and 30 days after exposure to the different photoperiods. Additionally, we organized the plants in intervals of plant height and choose three representative plants regarding those intervals, from each diel condition to access their roots, leaves and leaf sheaths dry masses at day 0; and 3 other plants at day 30 for the same measurements.

### Isolation and quantification of cell wall components

Three biological replicates of 300 mg from pools of leaves and leaf sheaths were used to determine pectin, hemicellulose and cellulose in technical triplicates in each tissue and in each harvested ZT following the method described previously [66].

For the pectin fraction, samples were washed and centrifuged at 13,000 RPM for 10 min with 1 mL chilled water, 1 mL acetone and 1 mL of a 1:1 (v/v) methanol-chloroform solution. Pellets were dried at room temperature and then incubated for 3 h at 37 °C in 1 mL of a α-amylase solution (2 U/mL) in 0.1 M sodium acetate buffer (pH 6.5). Samples were centrifuged at 13,000 RPM for 10 min, and the pellets were incubated three times in 600 μL of 20 mM ammonium oxalate (pH 4) for 1 h at 70 °C. The supernatants were collected after centrifugation and combined in the same microtube. The pellets resulting from this extraction were used to extract the hemicellulose fraction. They were incubated with 600 μL of 0.1 M NaOH for 24 h under vacuum at room temperature in the dark. Samples were centrifuged at 13,000 RPM for 10 min, and supernatants were collected. Pellets were then incubated three times with 400 μL of 17.5% (w/v) NaOH for 8 h in the same conditions. The four supernatants obtained by centrifugation were combined in the same microtube. Finally, the cellulosic fraction was extracted. Pellets were washed with the following solutions (1 mL): water; 1 mM acetic acid; 1 mL ethanol. After drying at room temperature, the pellets were resuspended in 1 mL 72% (v/v) H_2_SO_4_ for 1 h, with vortexing every 10 min. The sugar content from each fraction was quantified in triplicate following the phenol-sulfuric method [67], using glucose as standard.

### Extraction and quantification of soluble sugars

Leaves and leaf sheaths were ground in liquid nitrogen in triplicate in the same conditions as described in the previous section. Total soluble sugars, sucrose and reducing sugars were each extracted from samples of 20 mg of freeze-dried material. Samples were extracted with 1.5 mL 70% (v/v) ethanol at 70 °C for 1 h and centrifuged at 13,000 RPM for 10 min. This procedure was repeated four times, and the solutions were pooled in the same microtube [49]. Sucrose and total sugar contents were quantified in technical triplicates with the phenol-sulfuric method [67], using pure sucrose and glucose to build standard curves. For sucrose content, solutions were boiled in presence of 30% KOH for 10 min before measurements. Reducing sugars quantification was performed according to the Somogyi-Nelson protocol [68], with glucose as reference.

### Statistical analysis

Significantly different means from sugars measurements were assessed through Student’s two-sided unpaired t-test using the “*t-test”* function in R. *P*-values < 0.05 were considered significant. ANOVA was used to access differences within size and dry mass measurements.

## Additional files

Additional file 1: **Figure S1**. Schematic overview of the time points accordingly to each photoperiod. (PDF 59 kb)

Additional file 2: **Table S1**. Physiological measurements from plants under 12 h/12 h and 08 h/16 h light/dark regimes. **Table S2**. ANOVA tables from comparisons between + 1 leaf length, width and plant height in days 0 and 30. **Table S3.** Dry mass measurements made at days 0 and 30. **Table S4.** ANOVA tables for data from dry mass measurements made at days 0 and 30. **Table S5.** Cell wall components measurements accordingly to each ZT and photoperiod. **Table S6.** Significant differences (*p*-value < 0.05) between time points for each measured cell wall components in the leaf and leaf sheath in plants under 12 h/12 h and 08 h/16 h photoperiods. **Table S7.** Soluble sugars measurements accordingly to each ZT and photoperiod. **Table S8.** Significant differences (p-value < 0.05) between time points for each measured soluble sugar in the leaf and leaf sheath in plants under 12 h/12 h and 08 h/16 h photoperiods (*Student’s t-test). **Table S9.** Sugars quantification data from sugarcane leaves and leaf sheaths in mg/g*. (PDF 315 kb)

## Data Availability

The datasets generated or analysed during this study are included in this published article and its additional files and are available from the corresponding author on reasonable request.

## Abbreviations

2G ethanol: Second generation ethanol
GH: Glucosyl hydrolases
INV: Invertases
L/D: Light/dark
OR: Oxido-reductases
RD: Regular day
S6P: Sucrose-6-phosphate
SD: Short-day
SnRK: Sucrose nonfermenting related kinase
ZT: Zeitgeber time
