# Supplementary figures and images for: Diel oscillations in cell wall components and soluble sugars as a response to short-day in sugarcane (Saccharum sp.)
Source: BMC Plant Biol. 2019 May 23;19:215. doi: 10.1186/s12870-019-1837-4 (PMC6533765; doi:10.1186/s12870-019-1837-4)

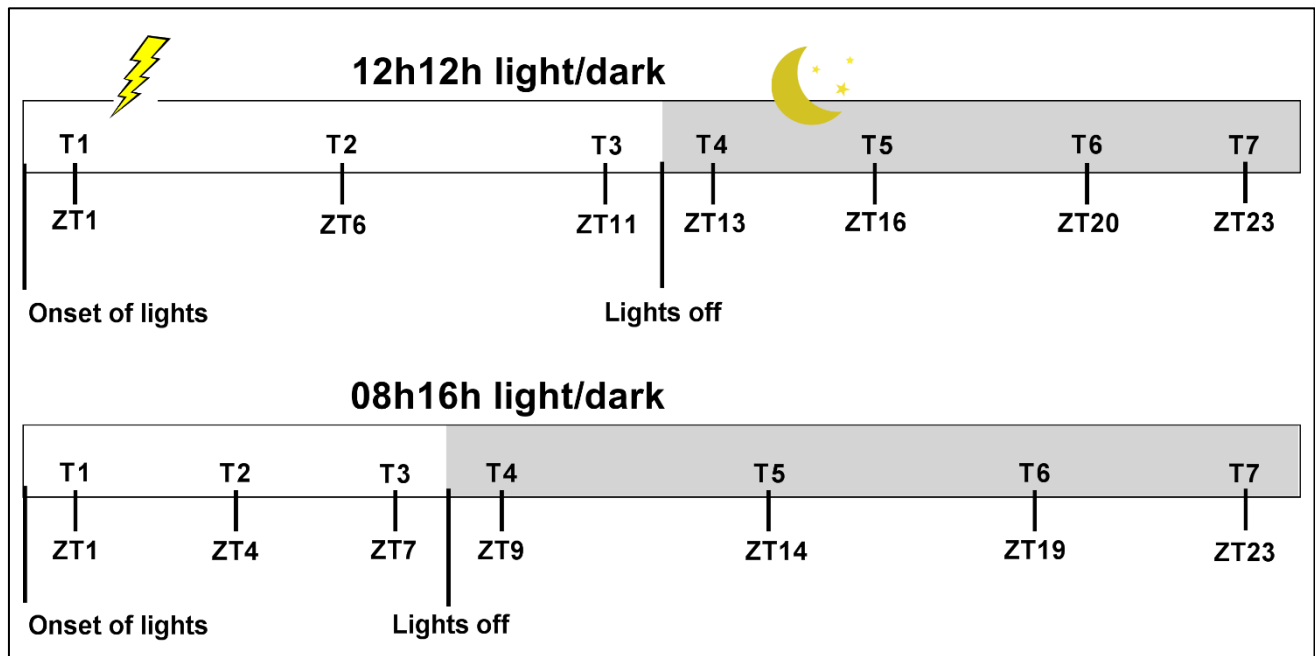

Supplement: Supplementary file 1 — Figure S1. Schematic overview of the time points accordingly to each photoperiod. (PDF 59 kb) [file 12870_2019_1837_MOESM1_ESM.pdf]
