# Supplementary material for: Diel oscillations in cell wall components and soluble sugars as a response to short-day in sugarcane (Saccharum sp.)
Source: BMC Plant Biol. 2019 May 23;19:215. doi: 10.1186/s12870-019-1837-4 (PMC6533765; doi:10.1186/s12870-019-1837-4)
Supplement: Supplementary file 2 — Table S1. Physiological measurements from plants under 12 h/12 h and 08 h/16 h light/dark regimes. Table S2. ANOVA tables from comparisons between + 1 leaf length, width and plant height in days 0 and 30. Table S3. Dry mass measurements made at days 0 and 30. Table S4. ANOVA tables for data from dry mass measurements made at days 0 and 30. Table S5. Cell wall components measurements accordingly to each ZT and photoperiod. Table S6. Significant differences (p-value < 0.05) between time points for each measured cell wall components in the leaf and leaf sheath in plants under 12 h/12 h and 08 h/16 h photoperiods. Table S7. Soluble sugars measurements accordingly to each ZT and photoperiod. Table S8. Significant differences (p-value < 0.05) between time points for each measured soluble sugar in the leaf and leaf sheath in plants under 12 h/12 h and 08 h/16 h photoperiods (*Student’s t-test). Table S9. Sugars quantification data from sugarcane leaves and leaf sheaths in mg/g*. (PDF 315 kb) [file 12870_2019_1837_MOESM2_ESM.pdf]

Additional file 2: Table S1. Physiological measurements from plants under 12h/12h and 08h/16h light/dark regimes. Twenty-seven random plants from each population were chosen for accessing +1 leaves length and width; and plant height in days 0 and 30.

Day 0							
12h/12h light/dark				08h/16h light/dark			
+1 leaf			Plant height (cm)	+1 leaf			Plant height (cm)
Plant id	Length (cm)	Width (cm)		Plant id	Length (cm)	Width (cm)	
1	33.0	1.0	41.2	1	40.1	1.0	70.2
3	21.3	0.9	46.0	2	47.0	1.2	58.5
4	16.9	1.2	38.5	3	23.0	1.1	32.0
5	27.2	1.0	34.3	5	17.1	1.4	39.2
6	30.2	0.6	40.0	6	39.5	1.2	58.5
7	26.7	1.1	45.6	7	34.0	0.8	47.0
8	40.1	1.0	53.5	8	18.5	1.0	33.0
9	50.3	1.2	62.5	9	51.0	1.4	64.0
10	43.8	1.2	57.5	10	41.0	1.1	63.0
12	46.0	1.3	67.0	12	52.0	1.7	64.0
13	37.2	1.0	64.0	13	57.0	1.4	97.0
14	29.8	1.1	39.0	14	29.5	1.4	69.8
15	57.3	1.3	81.9	15	40.0	1.3	59.5
16	43.6	0.6	56.0	16	32.8	1.4	49.5
17	38.2	0.7	60.4	17	17.1	0.8	33.5
18	64.0	1.5	80.2	18	22.8	1.1	38.9
20	54.5	1.3	67.9	19	48.5	1.4	75.0
21	24.0	1.2	33.0	20	38.9	1.2	72.6
22	42.2	1.1	65.4	21	36.1	1.2	51.2
23	20.5	0.9	32.4	23	54.2	1.3	70.0
24	18.9	0.9	29.3	24	30.2	0.9	44.2
25	44.2	1.4	44.2	25	29.8	1.4	43.9
26	53.0	1.4	77.2	26	26.9	0.9	55.4
27	32.1	1.4	47.8	27	44.8	1.2	54.8
28	43.1	1.0	55.0	28	60.2	1.2	78.0
29	51.2	1.1	68.1	29	51.0	1.0	65.7
30	31.8	1.0	55.1	30	29.2	0.9	29.2
Average	37.8	1.1	53.4	Average	37.5	1.2	56.2

Table S1 cont.:

Day 30							
12h/12h light/dark				08h/16h light/dark			
+1 leaf			Plant height (cm)	+1 leaf			Plant height (cm)
Plant id	Length (cm)	Width (cm)		Plant id	Length (cm)	Width (cm)	
1	33.1	0.9	49.6	1	39.8	1.0	110.4
3	21.0	0.9	79.4	2	46.3	1.3	102.0
4	17.1	1.2	49.0	3	23.1	1.1	51.4
5	27.5	1.0	41.9	5	16.9	1.1	49.5
6	30.8	0.6	75.0	6	39.2	1.2	82.1
7	27.9	1.1	49.1	7	34.0	0.8	83.2
8	41.2	1.0	77.5	8	50.0	1.4	125.0
9	50.4	1.2	109.6	9	51.2	1.3	106.0
10	44.1	1.1	103.5	10	41.6	1.1	101.0
12	46.1	1.2	104.2	12	52.1	1.8	104.2
13	38.9	1.1	105.6	13	57.2	1.4	134.0
14	29.8	1.1	67.4	14	40.0	1.5	116.7
15	57.6	1.2	121.0	15	39.0	1.3	87.0
16	44.2	0.9	91.2	16	33.9	1.4	70.2
17	38.8	0.9	98.0	17	17.8	0.8	38.9
18	63.9	1.5	121.0	18	22.0	1.1	60.0
20	54.6	1.3	105.5	19	48.1	1.4	114.0
21	26.3	1.2	47.0	20	39.1	1.2	114.6
22	42.5	1.1	116.6	21	35.6	1.2	90.2
23	20.6	0.9	57.1	23	54.2	1.4	126.0
24	18.6	0.9	48.5	24	30.1	1.0	67.6
25	44.5	1.4	100.3	25	30.0	1.3	71.6
26	53.0	1.3	111.0	26	26.5	1.0	84.2
27	31.8	1.4	74.2	27	45.1	1.2	97.0
28	43.2	1.0	92.9	28	60.5	1.2	128.0
29	50.7	1.1	106.8	29	45.0	1.1	87.5
30	32.5	1.1	93.0	30	29.1	0.9	73.1
Average	38.2	1.1	85.0	Average	38.8	1.2	91.7

Additional file 2: Table S2. ANOVA tables from comparisons between +1 leaf length, width and plant height in days 0 and 30.

+1 leaf length day 0						
Anova: Single Factor						
SUMMARY						
Groups	*Count*	*Sum*	*Average*	*Variance*		
12h/12h	27	1021.1	37.8185	159.553		
08h/16h	27	1012.2	37.4889	156.105		
ANOVA						
Source of Variation	*SS*	*df*	*MS*	*F*	*P-value*	*F crit*
Between Groups	1.46685	1	1.46685	0.00929	0.92357	4.02663
Within Groups	8207.11	52	157.829			
Total	8208.57	53				

+1 leaf width day 0						
Anova: Single Factor						
SUMMARY						
Groups	*Count*	*Sum*	*Average*	*Variance*		
12h/12h	27	29.4	1.08889	0.05487		
08h/16h	27	31.9	1.18148	0.04926		
ANOVA						
Source of Variation	*SS*	*df*	*MS*	*F*	*P-value*	*F crit*
Between Groups	0.11574	1	0.11574	2.22298	0.14201	4.02663
Within Groups	2.70741	52	0.05207			
Total	2.82315	53				

Total plant height day 0						
Anova: Single Factor						
SUMMARY						
Groups	*Count*	*Sum*	*Average*	*Variance*		
12h/12h	27	1443	53.4444	227.597		
08h/16h	27	1517.6	56.2074	270.085		
ANOVA						
Source of Variation	*SS*	*df*	*MS*	*F*	*P-value*	*F crit*
Between Groups	103.059	1	103.059	0.41415	0.5227	4.02663
Within Groups	12939.7	52	248.841			
Total	13042.8	53				

Table S2 cont.:

+1 leaf length day 30						
Anova: Single Factor						
SUMMARY						
Groups	*Count*	*Sum*	*Average*	*Variance*		
12h/12h	27	1030.7	38.1741	156.85		
08h/16h	27	1047.4	38.7926	139.972		
ANOVA						
Source of Variation	*SS*	*df*	*MS*	*F*	*P-value*	*F crit*
Between Groups	5.16463	1	5.16463	0.0348	0.85274	4.02663
Within Groups	7717.39	52	148.411			
Total	7722.56	53				

+1 leaf width day 30						
Anova: Single Factor						
SUMMARY						
Groups	*Count*	*Sum*	*Average*	*Variance*		
12h/12h	27	29.6	1.0963	0.03806		
08h/16h	27	32.5	1.2037	0.04883		
ANOVA						
Source of Variation	*SS*	*df*	*MS*	*F*	*P-value*	*F crit*
Between Groups	0.15574	1	0.15574	3.58459	0.06389	4.02663
Within Groups	2.25926	52	0.04345			
Total	2.415	53				

Total plant heigth day 30						
Anova: Single Factor						
SUMMARY						
Groups	*Count*	*Sum*	*Average*	*Variance*		
12h/12h	27	2295.9	85.0333	666.716		
08h/16h	27	2475.4	91.6815	662.442		
ANOVA						
Source of Variation	*SS*	*df*	*MS*	*F*	*P-value*	*F crit*
Between Groups	596.671	1	596.671	0.89782	0.34775	4.02663
Within Groups	34558.1	52	664.579			
Total	35154.8	53				

Additional file 2: Table S3. Dry mass measurements made at days 0 and 30. Plants were organized in intervals regarding the plant height and three representative plants were harvested from each diel condition to access root, leaves and leaf sheaths dry masses at day 0; and three other plants at day 30 for the same measurements.

12h/12h light/dark						08h/16h light/dark				
Dry mass						Dry mass				
Plant	Leaves (g)	Leaf sheath (g)	Roots (g)	Total (g)		Plant	Leaves (g)	Leaf sheath (g)	Roots (g)	Total (g)
Day 0						Day 0				
1	0.58	0.73	0.42	1.73		1	0.35	0.51	0.25	1.11
2	0.18	0.24	0.25	0.67		2	0.84	0.97	0.71	2.52
3	0.84	1.17	1.14	3.15		3	0.10	0.25	0.22	0.57
Day 30						Day 30				
1	0.40	0.21	0.07	0.68		1	0.32	0.57	0.25	1.14
2	1.10	1.16	0.78	3.04		2	1.04	0.98	0.69	2.70
3	1.52	1.46	0.86	3.84		3	1.33	1.02	0.44	2.79

Additional file 2: Table S4. ANOVA tables for data from dry mass measurements made at days 0 and 30.

Leaves day 0						
Anova: Single Factor						
SUMMARY						
Groups	Count	Sum	Average	Variance		
Leaves 12h/12h	3	1.6	0.532	0.109459		
Leaves 08h/16h	3	1.3	0.43166667	0.14227233		
ANOVA						
Source of Variation	SS	df	MS	F	P-value	F crit
Between Groups	0.01510017	1	0.01510017	0.1199705	0.74652	7.70865
Within Groups	0.50346267	4	0.12586567			
Total	0.51856283	5				
Leaf sheaths day 0						
Anova: Single Factor						
SUMMARY						
Groups	Count	Sum	Average	Variance		
Leaf sheaths 12h/12h	3	2.15	0.71533333	0.21643333		
Leaf sheaths 08h/16h	3	1.73	0.577	0.132303		
ANOVA						
Source of Variation	SS	df	MS	F	P-value	F crit
Between Groups	0.02870417	1	0.02870417	0.16461816	0.70571	7.70865
Within Groups	0.69747267	4	0.17436817			
Total	0.72617683	5				
Roots day 0						
Anova: Single Factor						
SUMMARY						
Groups	Count	Sum	Average	Variance		
Roots 12h/12h	3	1.81	0.60366667	0.22504933		
Roots 08h/16h	3	1.18	0.39166667	0.07584933		
ANOVA						
Source of Variation	SS	df	MS	F	P-value	F crit
Between Groups	0.067416	1	0.067416	0.4480977	0.5399	7.70865
Within Groups	0.60179733	4	0.15044933			
Total	0.66921333	5				

Table S4 cont.:

Total dry weight day 0						
Anova: Single Factor						
Total day 0						
SUMMARY						
Groups	Count	Sum	Average	Variance		
total 12h	3	5.55	1.851	1.545921		
total 08h	3	4.2	1.40033333	1.01710933		
ANOVA						
Source of Variation	SS	df	MS	F	P-value	F crit
Between Groups	0.30465067	1	0.30465067	0.23772693	0.65137	7.70865
Within Groups	5.12606067	4	1.28151517			
Total	5.43071133	5				

Leaves day 30						
Anova: Single Factor						
SUMMARY						
Groups	Count	Sum	Average	Variance		
Leaves 12h/12h	3	3.02	1.00633333	0.31850233		
Leaves 08h/16h	3	2.69	0.89633333	0.27218633		
ANOVA						
Source of Variation	SS	df	MS	F	P-value	F crit
Between Groups	0.01815	1	0.01815	0.06145369	0.81642	7.70865
Within Groups	1.18137733	4	0.29534433			
Total	1.19952733	5				

Leaf sheaths day 30						
Anova: Single Factor						
SUMMARY						
Groups	Count	Sum	Average	Variance		
Leaf sheaths 12h/12h	3	2.83	0.94233333	0.42678433		
Leaf sheaths 08h/16h	3	2.56	0.85433333	0.06101433		
ANOVA						
Source of Variation	SS	df	MS	F	P-value	F crit
Between Groups	0.011616	1	0.011616	0.04762621	0.83793	7.70865
Within Groups	0.97559733	4	0.24389933			
Total	0.98721333	5				

Table S4 cont.:

Roots day 30						
Anova: Single Factor						
SUMMARY						
Groups	Count	Sum	Average	Variance		
Roots 12h/12h	3	1.72	0.57266667	0.18960033		
Roots 08h/16h	3	1.39	0.46166667	0.04841433		
ANOVA						
Source of Variation	SS	df	MS	F	P-value	F crit
Between Groups	0.0184815	1	0.0184815	0.15529715	0.71363	7.70865
Within Groups	0.47602933	4	0.11900733			
Total	0.49451083	5				

Total dry weight day 30						
Anova: Single Factor						
Total day 30						
SUMMARY						
Groups	Count	Sum	Average	Variance		
total 12h	3	7.56	2.52133333	2.69892233		
total 08h	3	6.64	2.21233333	0.86449433		
ANOVA						
Source of Variation	SS	df	MS	F	P-value	F crit
Between Groups	0.1432215	1	0.1432215	0.08038437	0.79085	7.70865
Within Groups	7.12683333	4	1.78170833			
Total	7.27005483	5				

Additional file 2: Table S5. Cell wall components measurements accordingly to each ZT and photoperiod. Values are mean ± SD, n=3.

Cell wall components (mg/g Dry mass^{-1})							
Photoperiod	Timepoint	Pectin		Hemicelluloses		Celluloses	
		Leaf	Leaf sheath	Leaf	Leaf sheath	Leaf	Leaf sheath
12h12h	ZT1	4.94 ± 0.14	4.84 ± 1.10	269.40 ± 24.88	251.76 ± 16.16	394.06 ± 17.32	456.56 ± 24.68
	ZT6	4.66 ± 0.75	5.06 ± 1.72	207.97 ± 51.93	252.11 ± 4.54	398.06 ± 76.66	522.45 ± 41.87
	ZT11	5.48 ± 1.20	3.91 ± 0.35	211.72 ± 51.50	244.89 ± 9.78	373.58 ± 73.90	522.42 ± 26.84
	ZT13	5.67 ± 0.56	4.21 ± 0.36	255.43 ± 46.63	259.75 ± 10.33	343.83 ± 44.67	499.35 ± 37.74
	ZT16	4.71 ± 0.24	5.45 ± 0.49	261.87 ± 5.75	284.19 ± 33.25	370.45 ± 27.53	575.92 ± 41.23
	ZT20	4.53 ± 0.91	3.67 ± 0.38	222.28 ± 54.89	218.93 ± 22.15	382.21 ± 105.89	508.79 ± 9.62
	ZT23	4.01 ± 0.70	3.39 ± 0.54	197.58 ± 47.08	235.71 ± 15.88	343.93 ± 110.34	528.39 ± 53.3
08h16h	ZT1	4.04 ± 0.20	4.32 ± 0.80	260.37 ± 25.66	235.91 ± 29.00	418.19 ± 33.60	444.20 ± 56.18
	ZT4	5.86 ± 0.52	4.98 ± 0.74	191.01 ± 30.11	220.98 ± 12.79	335.48 ± 68.40	438.15 ± 20.54
	ZT7	5.17 ± 1.04	4.57 ± 0.39	273.86 ± 16.01	266.36 ± 14.51	407.26 ± 50.9	512.73 ± 49.33
	ZT9	4.02 ± 0.29	5.10 ± 0.46	211.67 ± 22.12	271.94 ± 13.46	320.33 ± 23.77	563.54 ± 35.24
	ZT14	4.15 ± 1.30	6.07 ± 0.64	178.18 ± 24.04	261.47 ± 21.88	311.22 ± 82.82	563.09 ± 62.97
	ZT19	5.91 ± 0.44	5.01 ± 0.05	244.44 ± 33.80	232.54 ± 5.41	454.04 ± 55.90	416.85 ± 36.67
	ZT23	5.20 ± 1.18	4.51 ± 0.22	217.71 ± 23.43	289.81 ± 7.54	351.19 ± 79.05	566.40 ± 33.37

Additional file 2: Table 6. Significant differences (p-value < 0.05) between time points for each measured cell wall components in the leaf and leaf sheath in plants under 12h12h and 08h16h photoperiods.

Photoperiod	Cell wall fraction	Comparison	p-value (Student's t-test)
12h12h	Leaf sheath pectin	ZT11 vs ZT16	0.0267
		ZT13 vs ZT16	0.0492
		ZT16 vs ZT20	0.0173
		ZT16 vs ZT23	0.0166
	Leaf sheath cellulose	ZT1 vs ZT16	0.0341
08h16h	Leaf pectin	ZT1 vs ZT4	0.0263
		ZT1 vs ZT19	0.0146
		ZT4 vs ZT9	0.0201
		ZT9 vs ZT19	0.0104
	Leaf hemicelluloses	ZT4 vs ZT7	0.0404
		ZT7 vs ZT9	0.0368
		ZT7 vs ZT14	0.0131
	Leaf cellulose	ZT1 vs ZT9	0.0331
	Leaf sheath hemicelluloses	ZT4 vs ZT7	0.0301
		ZT4 vs ZT9	0.0179
		ZT4 vs ZT23	0.0056
		ZT9 vs ZT19	0.0391
		ZT19 vs ZT23	0.0014
	Leaf sheath cellulose	ZT4 vs ZT9	0.0194
		ZT4 vs ZT23	0.0151
		ZT9 vs ZT19	0.0152
		ZT19 vs ZT23	0.0132

Additional file 2: Table S7. Soluble sugars measurements accordingly to each ZT and photoperiod. Values are mean ± SD, n=3.

		Soluble sugars (mg/g Dry mass^{-1})					
Photoperiod	Timepoint	Total sugars		Sucrose		Reducing sugars	
		Leaf	Leaf sheath	Leaf	Leaf sheath	Leaf	Leaf sheath
12h12h	ZT1	72.51 ± 14.64	100.84 ± 33.42	55.83 ± 4.83	67.65 ± 14.25	9.30 ± 1.48	10.88 ± 2.14
	ZT6	99.83 ± 13.69	113.96 ± 14.41	73.79 ± 16.03	82.45 ± 5.42	9.39 ± 3.42	15.23 ± 2.51
	ZT11	101.76 ± 9.98	108.51 ± 33.07	97.45 ± 6.10	80.01 ± 14.16	8.35 ± 0.48	11.56 ± 1.48
	ZT13	102.50 ± 13.68	170.12 ± 13.55	80.07 ± 8.46	124.27 ± 5.62	10.25 ± 1.22	11.57 ± 0.93
	ZT16	80.64 ± 1.87	157.16 ± 8.99	72.41 ± 2.19	88.87 ± 1.69	8.71 ± 1.56	10.87 ± 0.45
	ZT20	77.56 ± 1.38	186.19 ± 8.79	73.54 ± 3.29	103.34 ± 6.34	8.92 ± 0.34	18.13 ± 0.62
	ZT23	65.22 ± 4.27	113.35 ± 15.45	70.24 ± 8.18	61.27 ± 19.39	9.90 ± 2.40	8.47 ± 1.79
08h16h	ZT1	69.35 ± 4.71	66.84 ± 13.17	68.66 ± 3.89	43.23 ± 4.00	8.76 ± 1.89	5.54 ± 0.31
	ZT4	61.55 ± 2.67	75.62 ± 9.72	69.91 ± 5.21	46.61 ± 2.51	8.49 ± 0.37	7.47 ± 0.76
	ZT7	82.30 ± 8.33	76.59 ± 8.48	71.56 ± 5.27	53.09 ± 5.79	11.52 ± 1.13	8.55 ± 2.16
	ZT9	76.46 ± 4.54	97.82 ± 4.58	62.32 ± 3.06	67.50 ± 2.67	12.04 ± 0.98	11.84 ± 2.39
	ZT14	88.56 ± 6.12	80.26 ± 11.99	79.65 ± 9.61	66.16 ± 12.98	9.89 ± 0.74	9.83 ± 1.67
	ZT19	85.19 ± 3.99	103.96 ± 1.52	90.11 ± 7.11	78.00 ± 3.99	11.35 ± 1.99	13.05 ± 0.95
	ZT23	57.19 ± 2.81	68.11 ± 4.13	64.45 ± 2.26	51.42 ± 3.85	9.23 ± 0.28	14.21 ± 2.64

Additional file 2: Table S8. Significant differences (p-value < 0.05) between time points for each measured soluble sugar in the leaf and leaf sheath in plants under 12h12h and 08h16h photoperiods (*Student's t-test).

12h12h		
Fraction	Comparison	p-value*
Leaf total sugars	ZT11 vs ZT23	0.0221
	ZT16 vs ZT23	0.0225
	ZT20 vs ZT23	0.0442
Leaf sucrose	ZT1 vs ZT11	0.002
	ZT1 vs ZT13	0.0355
	ZT1 vs ZT16	0.025
	ZT1 vs ZT20	0.0167
	ZT11 vs ZT16	0.019
	ZT11 vs ZT20	0.0156
	ZT11 vs ZT23	0.0226
Leaf sheath total sugars	ZT6 vs ZT13	0.016
	ZT6 vs ZT16	0.0306
	ZT6 vs ZT20	0.0068
	ZT13 vs ZT23	0.018
	ZT16 vs ZT20	0.031
	ZT16 vs ZT23	0.0363
	ZT20 vs ZT23	0.0087
Leaf sheath sucrose	ZT1 vs ZT13	0.0191
	ZT6 vs ZT13	0.0016
	ZT6 vs ZT20	0.0249
	ZT11 vs ZT13	0.0338
	ZT13 vs ZT16	0.0079
	ZT13 vs ZT20	0.0256
	ZT13 vs ZT23	0.0357
Leaf sheath reducing sugars	ZT1 vs ZT20	0.0327
	ZT6 vs ZT23	0.0416
	ZT11 vs ZT20	0.0138
	ZT13 vs ZT20	0.002
	ZT16 vs ZT20	0.0003
	ZT20 vs ZT23	0.0101

8h16h		
Fraction	Comparison	p-value*
Leaf total sugars	ZT1 vs ZT14	0.0272
	ZT1 vs ZT19	0.0233
	ZT1 vs ZT23	0.0462
	ZT4 vs ZT9	0.0242
	ZT4 vs ZT14	0.0136
	ZT4 vs ZT19	0.0036
	ZT7 vs ZT23	0.0396
	ZT9 vs ZT23	0.0112
	ZT14 vs ZT23	0.0087
	ZT19 vs ZT23	0.0019
Leaf sucrose	ZT1 vs ZT19	0.0315
	ZT4 vs ZT19	0.0358
	ZT7 vs ZT19	0.0457
	ZT9 vs ZT19	0.0186
	ZT19 vs ZT23	0.0273
Leaf reducing sugars	ZT4 vs ZT9	0.0248
	ZT9 vs ZT23	0.0466
Leaf sheath total sugars	ZT7 vs ZT19	0.0411
	ZT9 vs ZT23	0.0025
	ZT19 vs ZT23	0.0029
Leaf sheath sucrose	ZT1 vs ZT9	0.0034
	ZT1 vs ZT19	0.0009
	ZT4 vs ZT9	0.0013
	ZT4 vs ZT19	0.0016
	ZT7 vs ZT19	0.0101
	ZT9 vs ZT19	0.0437
	ZT9 vs ZT23	0.011
	ZT19 vs ZT23	0.0025
Leaf sheath reducing sugars	ZT1 vs ZT19	0.0043
	ZT1 vs ZT23	0.0416
	ZT4 vs ZT19	0.0035

Additional file 2: Table S9. Sugars quantification data from sugarcane leaves and culms in mg/g*. Data shown stands for untreated samples only.

Sugar (mg/g)		Organ	Additional information	Reference
Sucrose	Reducing sugars**			
11.70	6.10	Leaf	Young leaves; mg/g FW***	Batta and Singh, 1986
28.70	19.10		3-month-old****; mg/g FW	Batta et al., 1995
10.27	1.35		4-month-old; 27°C; mg/g FW	Ebrahim et al., 1998
6.20	4.00		3-month-old; mg/g FW	Sachdeva et al., 2003
13.69	2.79		+3 leaf; 9-12-month-old; field; mg/g FW	McCormick et al., 2006
14.02	0.09		3-month-old; mg/g FW	Mattiello et al., 2015
33.40	5.9		9-month-old; +1 leaf; field	Ferreira et al., 2016
50.00	110.00		+2 leaf; 32 days old;	Cunha et al., 2017
29.20	50.40	Culm	Young; mid culm	Batta and Singh, 1986
134.00	26.00		6-month-old; mid culm; mg/g FW	Batta et al., 1995
102.69	*n/a*		9-month-old; range from 8.6 - 102.7	Zhu et al., 1997
85.75	10.81		4-month-old; mid culm; 27°C; mg/g FW	Ebrahim et al., 1998
400.00	*n/a*		adult; field; mid culm	Botha and Black, 2000
25.00	22.00		3-month-old; mid culm; mg/g FW	Sachdeva et al., 2003
17.11	20.71		9-12-month-old; internode 6; field; mg/g FW	McCormick et al., 2006
177.99	14.41		16-month-old; internode 7	Wu and Birch, 2007
150.00	20.00		7.5-month-old; upper culm; mg/g FW	Batta et al., 2008
38.00	*n/a*		10-month-old; young internodes; hot chamber	Imman-Bamber et al., 2010
34.50	147.9		9-month-old; mid internodes; field	Ferreira et al., 2016
190.00	210.00		32 days old; upper culm	Cunha et al., 2017

*data presented in μmol/g was converted to mg/g.
**data presented as glucose and fructose in the literature are shown as reducing sugars in a joined column
***FW stands for fresh weight.
****at this stage culms are leaf sheath but already accumulate sucrose.
